# Highly sensitive and specific diagnosis of COVID-19 by reverse transcription multiple cross-displacement amplification-labelled nanoparticles biosensor

**DOI:** 10.1183/13993003.02060-2020

**Published:** 2020-12-10

**Authors:** Shijun Li, Weijia Jiang, Junfei Huang, Ying Liu, Lijuan Ren, Li Zhuang, Qinni Zheng, Ming Wang, Rui Yang, Yi Zeng, Yi Wang

**Affiliations:** 1 Laboratory of Bacterial Infectious Disease of Experimental Center, Guizhou Provincial Center for Disease Control and Prevention, Guiyang, P.R. China; 2 Dept of Respiratory Disease, Beijing Pediatric Research Institute, Beijing Children's Hospital, Capital Medical University, National Center for Children's Health, Beijing, P.R. China; 3 Key Laboratory of Major Diseases in Children, Ministry of Education, Beijing Key Laboratory of Pediatric Respiratory Infection Disease, National Clinical Research Center for Respiratory Diseases, Beijing Children's Hospital, Capital Medical University, National Center for Children's Health, Beijing, P.R. China

## Abstract

**Background:**

The ongoing outbreak of the novel human coronavirus severe acute respiratory syndrome coronavirus 2 (SARS-CoV-2) (also known as 2019-nCoV) has become a global health concern. Rapid and easy-to-use diagnostic techniques are urgently needed to diagnose SARS-CoV-2 infection.

**Methods:**

We devised a reverse transcription multiple cross-displacement amplification (RT-MCDA) coupled with a nanoparticle-based biosensor assay (RT-MCDA-BS) for rapid, sensitive and specific diagnosis of coronavirus disease 2019 (COVID-19). Two primer sets were designed to target the open reading frame 1a/b and nucleoprotein gene of SARS-CoV-2. A total of 183 clinical samples, including 65 patients with COVID-19 infection and 118 patients with other pathogen infections were used to testify the assay's feasibility. Diagnosis results were reported visually using the biosensor.

**Findings:**

The assay designed was performed using a simple instrument which could maintain the reaction in a constant temperature at 64°C for only 35 min. The total COVID-19 RT-MCDA-BS test procedure could be finished within 1 h. The COVID-19 RT-MCDA-BS could detect down to five copies of target sequences. Among 65 clinical samples from the COVID-19 patients, 22 (33.8%) positive results were obtained from faeces, nasal, pharyngeal and anal swabs *via* COVID-19 RT-MCDA-BS assay, while real-time reverse transcription-PCR assay only detected 20 (30.7%) positive results in these samples. No positive results were obtained from clinical samples with non-COVID-19 infections.

**Interpretation:**

COVID-19 RT-MCDA-BS was a rapid, reliable, low-cost and easy-to-use assay, which could provide an attractive laboratory tool to diagnose COVID-19 in multiple clinical specimens, especially for field, clinic laboratories and primary care facilities in resource-poor settings.

## Abstract 2

**We devised a novel method (COVID-19 RT-MCDA-BS) to diagnose COVID-19. Its rapidity, low cost and ease of use make the method an ideal tool for use in field, primary and clinical laboratories, especially for resource-poor settings.** https://bit.ly/3j08CU2

## Introduction

In late December 2019, an outbreak of pneumonia caused by a novel human coronavirus (severe acute respiratory syndrome coronavirus 2 (SARS-CoV-2); also referred to 2019-nCov) emerged in Wuhan, in the Hubei province in China [1, 2]. The novel emerging virus has rapidly spread across China, and to >200 countries/regions outside of China [3, 4]. As of May 28, 2020, SARS-CoV-2 has affected >5 593 631 patients (353 334 deaths) worldwide [4]. Due to its rapid spreading speed (R_0_ 3.28), possible fatal progression and strong infectivity, coronavirus disease 2019 (COVID-19) has stirred grave global concern [5]. Hence, a rapid, easy-to-use and reliable test tool was urgently required for diagnosing infections, treating the patients and controlling the spreading of COVID-19.

Early diagnosis according only to manifestations of COVID-19, which are highly variable, is extremely difficult [6]. The clinical manifestations of COVID-19 range from asymptomatic infection, nonspecific flu-like clinical presentations (such as cough and fever) to respiratory failure [3]. Obvious clinical manifestations may occur in 2 days or up to 2 weeks after exposure. Thus, designing a rapid and early diagnostic method is vital for treatment and disease control. Currently, the definitive diagnosis of COVID-19 infections strongly relies on PCR-based methods, such as reverse transcription (RT)-PCR and real-time RT (rRT)-PCR [6]. However, they require expensive thermal cycle apparatus and very skilful laboratory workers. In addition, the 62% positive rate obtained by PCR-based technologies because of their sensitivity limitation among clinically diagnosed COVID-19 patients is not effective for infection control [6, 7]. Thus, an easy-to-use and cost-effective test with higher sensitivity is needed as a first-line technique to tackle the COVID-19 in field and clinical settings.

Recently, several loop-mediated isothermal amplification (LAMP) assays have been devised for rapid detection of COVID-19 in clinical and stimulated patient samples [8–10]. However, the COVID-19 LAMP assays only detect one target gene (open reading frame 1a/b (F1ab)). False-positive results could be produced when they were applied to detect clinical samples containing highly homologous sequences, such as genes from bat severe acute respiratory syndrome-like coronavirus (GenBank KY417152.1). In addition, LAMP results are reported *via* agarose gel electrophoresis, SYBR dyes or pH indicator. In particular, the electrophoresis is a tedious and time-consuming process, and the assessment of colour change with the unaided eye is potentially subjective in an ambiguous test caused by the low concentration of templates. Novel techniques, which could overcome these technical difficulties, are continuously demanded.

The multiple cross-displacement amplification (MCDA) technique is an easy-to-perform and reliable diagnostic assay, which amplifies the target nucleic acid under conditions (usually between 58°C and 69°C) within a short time (20–40 min; usually 30 min) [11]. Simple equipment (such as a water bath, a heating block, even a thermos cup) is sufficient for MCDA testing. The MCDA technique has been used for diagnosing various infectious diseases producing positive results from down to three copies of a target sequence, which has shown its rapidity, simplicity and high specificity [12, 13]. To achieve a simultaneous detection of multiple targets and rapid analysis of MCDA products, nanoparticle-based biosensor testing has been coupled with MCDA to achieve objective detection results. The biosensor assay is simple, easy to conduct and low cost, and does not need complex instruments and skilled personnel [14].

**FIGURE 1:**
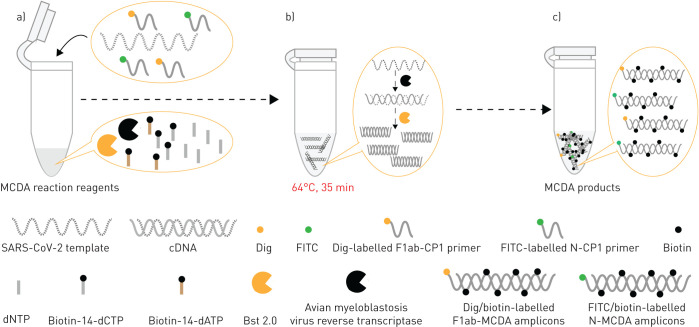
Mechanistic description of the coronavirus disease 2019 (COVID-19) reverse transcription multiple cross-displacement amplification nanoparticle-based biosensor assay (RT-MCDA-BS). a) Preparing the COVID-19 RT-MCDA reaction mixtures; b) one-step, single-tube reverse transcription MCDA reaction; c) the detectable COVID-19 RT-MCDA products are formed. SARS-CoV-2: severe acute respiratory syndrome coronavirus 2; dNTP: deoxynucleotide triphosphate; Dig: digoxigenin; FITC: fluorescein; F1ab: open reading frame 1a/b.

**FIGURE 2:**
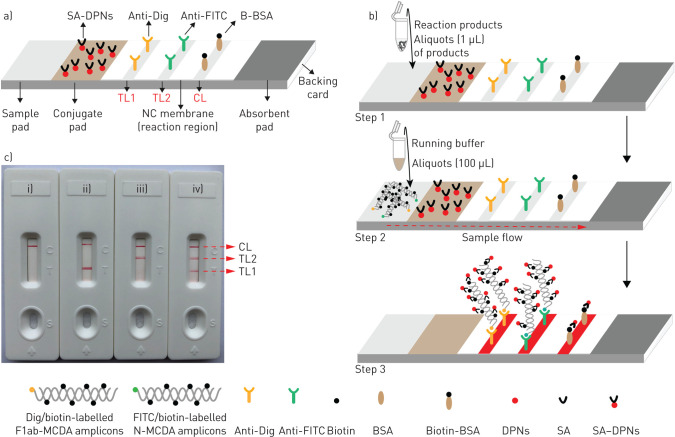
Schematic illustration of the principle of biosensor assay for visualisation of coronavirus disease 2019 (COVID-19) reverse transcription multiple cross-displacement amplification nanoparticle-based biosensor (RT-MCDA-BS) products. a) Biosensor detail; b) the test principle and steps of biosensor assay for COVID-19 RT-MCDA amplicons; c) interpretation of the detection results: i) negative (only the control line (CL) appears on the biosensor), ii) a positive result for open reading frame 1a/b (F1ab) (test line (TL)1 and CL appear on the detection region), iii) a positive result for N (TL2 and CL appear on the detection region), iv) a positive result for F1ab and nucleoprotein (N) (TL1, TL2 and CL appear on the biosensor). SA: streptavidin; DPNs: dye (crimson red)-coated polymer nanoparticles; anti-Dig: sheep anti-digoxigenin antibody; FITC: fluorescein; BSA: bovine serum albumin; NC: nitrocellulose.

We devised a one-step, single-tube reverse transcription MCDA (RT-MCDA) coupled with nanoparticle-based biosensor assay (RT-MCDA-BS) (figures 1 and 2), which could diagnose COVID-19 by simultaneously detecting F1ab and nucleoprotein (N) genes. The whole detection process could be completed with high accuracy within 1 h. Thus, the RT-MCDA-BS method may enable the diagnosis of COVID-19 easier, faster and more reliably, even in resource-limited settings.

## Methods

### Preparation of the nanoparticle-based biosensor

The nanoparticle-based biosensor (4×60 mm), depicted in figure 2a, consisted of a sample pad, a conjugate pad, a nitrocellulose (NC) membrane with an absorbent pad assembled on a plastic adhesive backing card (Jie-Yi Biotechnology, Shanghai, China). The capture reagents, including sheep anti-digoxigenin antibody (anti-Dig, 0.25 mg·mL^−1^; Abcam, Cambridge, UK), rabbit anti-fluorescein antibody (anti-FITC, 0.2 mg·mL^−1^; Abcam) and biotinylated bovine serum albumin (biotin-BSA, 4 mg·mL^−1^; Abcam), were dispensed onto the NC membrane as test line 1 (TL1), test line 2 (TL2) and control line (CL), respectively, with each line separated by 5 mm. The detector reagents (streptavidin–dye-coated polymer nanoparticles (SA–DPNs), 129 nm, 10 mg·mL^−1^; 100 mM borate, pH 8.5 with 0.1% BSA, 0.05% Tween 20 and 10 mM EDTA) were sprayed onto the conjugate region of the biosensor. Thus, the biosensor can detect three targets, including two target products (F1ab-MCDA amplicons and N-MCDA amplicons) and a chromatography control. Finally, the assembled biosensors were cut into 4-mm dipsticks, and dry-stored at room temperature for use.

### RT-MCDA primer design

**FIGURE 3:**
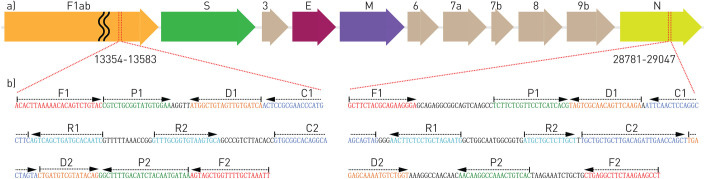
Primer design of coronavirus disease 2019 (COVID-19) reverse transcription multiple cross-displacement amplification nanoparticle-based biosensor (RT-MCDA-BS). a) Severe acute respiratory syndrome coronavirus 2 genome organisation (GenBank: MN908947, Wuhan-Hu-1); the length of genes are not drawn to scale. b) nucleotide sequence and location of open reading frame 1a/b (F1ab) and nucleoprotein (N) genes used to design the COVID-19 RT-MCDA primers. Part of the nucleotide sequences of F1ab and N are shown. The sites of primer sequence are marked in different colours. Right and left arrows show the sense and complementary sequences that are used. S: spike protein; E: envelope protein; M: membrane protein; 3, 6, 7a, 7b, 9b: accessory proteins.

Two sets of RT-MCDA primers (F1ab-MCDA and N-MCDA primer sets), which targeted the F1ab gene and N gene, respectively, of SARS-CoV-2 (GenBank MN908947, Wuhan-Hu-1), were designed according the MCDA principle (figure 3). Each MCDA primer set consists of two displacement primers (F1 and F2), two cross-primers (CP1 and CP2), and six amplification primers (C1, D1, R1, C2, D2 and R2). The specificity of the F1ab- and N-MCDA primer sets were analysed using the National Center for Biotechnology Information basic local alignment search tool. Moreover, OligoAnalyzer online software (V3.1; Integrated DNA Technologies, Coralville, IA, USA) was employed for primer, dimer and secondary structure investigation. The details of RT-MCDA primer design, primer location, sequences and modifications are listed in figure 3 and supplementary table S1. All oligomers were synthesised and purified by Tianyi-Huiyuan Biotech (Beijing, China) at HPLC purification grade.

### RT-MCDA reaction

The standard RT-MCDA (F1ab-MCDA/N-MCDA) was conducted in a one-step reaction in a 25-μL mixture containing 12.5 μL 2× isothermal reaction buffer (40 mM Tris-HCl (pH 8.8), 40 mM KCl, 16 mM MgSO_4_, 20 mM (NH4)_2_SO_4_, 2 M betaine and 0.2% Tween-20) (HuiDeXin Bio-technique, Tianjin, China); 8 U Bst 2.0 DNA polymerase (New England Biolabs; Ipswich, MA, USA); 10 U avian myeloblastosis virus reverse transcriptase (Invitrogen; Waltham, MA, USA); 1.4 mM dATP; 1.4 mM dCTP; 1.4 mM dGTP; 1.4 mM dTTP; 0.1 mM biotin-14-dCTP; 0.1 mM biotin-14-dATP; 0.4 μM each of displacement primers F1 and F2; 0.8 μM each of amplification primers C1, C2, D1, D1, R1 and R2; 0.8 μM each of cross-primers CP1* and CP1; 1.6 μM cross-primer CP2; and template (1 μL for the standard plasmid).

In addition, the COVID-19 RT-MCDA was performed in a one-step reaction in a 25-μL mixture containing 12.5 μL 2× isothermal reaction buffer (40 mM Tris-HCl (pH 8.8), 40 mM KCl, 16 mM MgSO_4_, 20 mM (NH4)_2_SO_4_, 2 M betaine and 0.2% Tween-20) (HuiDeXin Bio-technique); 8 U Bst 2.0 DNA polymerase (New England Biolabs); 10 U of the avian myeloblastosis virus reverse transcriptase (Invitrogen); 1.4 mM dATP; 1.4 mM dCTP; 1.4 mM dGTP; 1.4 mM dTTP; 0.1 mM biotin-14-dCTP; 0.1 mM biotin-14-dATP; 0.275 μM of F1ab-F1 and F1ab-F2; 0.55 μM of F1ab-C1, F1ab-C2, F1ab-D1, F1ab-D2, F1ab-R1 and F1ab-R2; 0.55 μM of F1ab-CP1* and CP1; 1.1 μM of F1ab-CP2; 0.125 μM of N-F1 and N-F2; 0.25 μM of N-C1, N-C2, N-D1, N-D2, N-R1 and N-R2; 0.25 μM of N-CP1* and CP1; 0.5 μM of N-CP2; and template (1 μL for the each standard plasmid, 5 μL for samples).

Real-time turbidity (LA-320C), visual detection reagents (VDR) and biosensors were applied to demonstrate the MCDA reactions and confirm the optimal amplification temperature.

### Sensitivity of the RT-MCDA-BS assay

Two plasmids (F1ab-plasmid and N-plasmid), which contain the F1ab and N genes, respectively, were constructed commercially by Tianyi-Huiyuan Biotech (Beijing, China). According to the manufacturer's manual, the initial concentrations of F1ab- and N-plasmids were 5×10^8^ copies·µL^−1^. Then, 10-fold serial dilutions (5×10^4^ to 5×10^−2^ copies) of F1ab-plasmid and N-plasmid were used to assess the limit of detection (LoD) of the COVID-19 RT-MCDA, F1ab-RT-MCDA and N-RT-MCDA assays. The examinations were conducted independently in triplicate. Simultaneously, the concentration of plasmid at the LoD level was used for confirming the optimal isothermal time of the COVID-19 RT-MCDA assay.

### Specificity of the COVID-19 RT-MCDA-BS assay

The analytical specificity of the COVID-19 RT-MCDA-BS assay was evaluated by comparing COVID-19 templates with the templates extracted from various viruses, bacteria and fungi (supplementary table S2).

### Validating the feasibility of COVID-19 RT-MCDA-BS using clinical samples

A total of 65 clinical samples (including faeces and anal, nasal and pharyngeal swabs) were obtained from patients in the acute phase and convalescence of COVID-19 sent to the Guizhou province centre for disease control (CDC) (supplementary table S3). Analysis of these RNA templates using the COVID-19 RT-MCDA-BS assay was approved by the Guizhou province CDC. The pharyngeal, nasal and anal swabs samples were collected using a flocked sterile plastic swab applicator, which was placed in a universal viral transport medium (UVIM) for viruses (HiDNA; YIMi Biotech, Taizhou, Jiangsu, China). For the faecal samples, aliquots (∼1 g) of the stools were placed into a tube, which contained 1.5 mL UVIM. Then, the faecal sample was centrifuged at 5000×*g* for 10 min, and the supernatant was placed into a new tube. Aliquots (200 µL) of UVIM (pharyngeal, nasal and anal swab samples) and supernatant of faecal samples were subjected to RNA template extraction procedure, which only required 15 min using a rapid RNA Extraction Kit (TianLong Biotech, Hangzhou, China). Aliquots of 5 µL of templates were used for conducting the RT-PCR and COVID-19 RT-MCDA-BS methods. The RNA templates extracted from different types of clinical samples were collected after rRT-PCR performance in the Guizhou province CDC, which was conducted using the officially approved clinical RT-PCR kit. The LoD of the RT-PCR diagnosis kit was 500 copies·mL^−1^ according its manual. A cycle threshold (Ct) value of <37 is defined as a positive result for COVID-19 infection; a Ct value of >40, or if no Ct value is obtained is defined as a negative result for COVID-19 infection; a Ct value between 37–40 is indeterminate, and the test should be examined again. In addition, 118 pharyngeal swabs samples collected from non-COVID-19 patients also were tested for validating the specificity of the COVID-19 RT-MCDA-BS method.

## Results

### Schematic mechanism of the COVID-19 RT-MCDA-BS assay

In the COVID-19 RT-MCDA system, two core primers, including F1ab-CP1 and N-CP1, are labelled at the 5′ end with digoxigenin (Dig) and FITC, respectively. The new F1ab-CP1 and N-CP1 primers are termed F1ab-CP1* and N-CP1*. Simultaneously, two components (biotin-14-dCTP and biotin-14-dATP) are added into the COVID-19 RT-MCDA reaction mixtures (figure 1a). As shown in figure 1b, the SARS-CoV-2 templates (RNA) are first converted to cDNA with the supplement of avian myeloblastosis virus reverse transcriptase at the amplification temperature (64°C). Then, the cDNA serves as the template for MCDA amplification. The F1ab-CP1* and N-CP1* primers anneal to the target regions, and is extended by the displacement enzyme (*Bst* 2.0), thus biotin-14-dATP and biotin-14-dCTP are incorporated into the newly synthesised products. As a result, a plenty of detectable double-labelled products are formed with F1ab-MCDA amplicons simultaneously labelled with Dig and biotin, and N-MCDA amplicons for FITC and biotin (figure 1c).

### The principle of biosensor visualisation of COVID-19 RT-MCDA results

Details of the nanoparticle-based biosensor are shown in figure 2a. After amplification, aliquots (1 μL) of COVID-19 RT-MCDA products were deposited on the sample pad of the biosensor (figure 2b, step 1), followed by the addition of an aliquot (100 μL) of running buffer to the same region (figure 2b, step 2). The running buffer can move along the biosensor through capillary action, and then rehydrate the immobilised SA–DPNs in conjugate region. F1ab-MCDA amplicons were specifically captured by the anti-Dig fixed on TL1, and the N-MCDA amplicons captured by the anti-FITC immobilised in TL2. The biotins of COVID-19 MCDA products can bind SA–DPNs for visualisation. The excess SA–DPNs were captured by biotinylated BSA immobilised in CL, which evaluated the working condition of the biosensor (figure 2b, step 3). The interpretation of the COVID-19 RT-MCDA results *via* biosensor is displayed in figure 2c.

### Confirmation and analysis of F1ab-, N- and COVID-19 RT-MCDA products

Using VDR, the positive F1ab-, N- and COVID-19 RT-MCDA vessels were visualised by the unaided eye as light green, while the vessels of negative and blank controls remained colourless (supplementary figure S1, top row). In the biosensor, two red bands were seen in TL1 and CL in positive F1ab-RT-MCDA amplification (supplementary figure S1a, bottom row), TL2 and CL in positive N-RT-MCDA amplification (supplementary figure S1b, bottom row). Simultaneous red bands in TL1, TL2 and CL biosensor indicate a positive result of COVID-19 RT-MCDA amplification (supplementary figure S1c, bottom row). A red band in the CL only represents negative and blank controls (supplementary figure S1a–c, bottom row). These data demonstrate that F1ab- and N-MCDA primer sets, and COVID-19 RT-MCDA assay are feasible for target sequence detection. Then, the optimal reaction temperature of 64°C of COVID-19 RT-MCDA was also determined (supplementary figure S2 and S3).

### Sensitivity of COVID-19 RT-MCDA-BS assay

**FIGURE 4:**
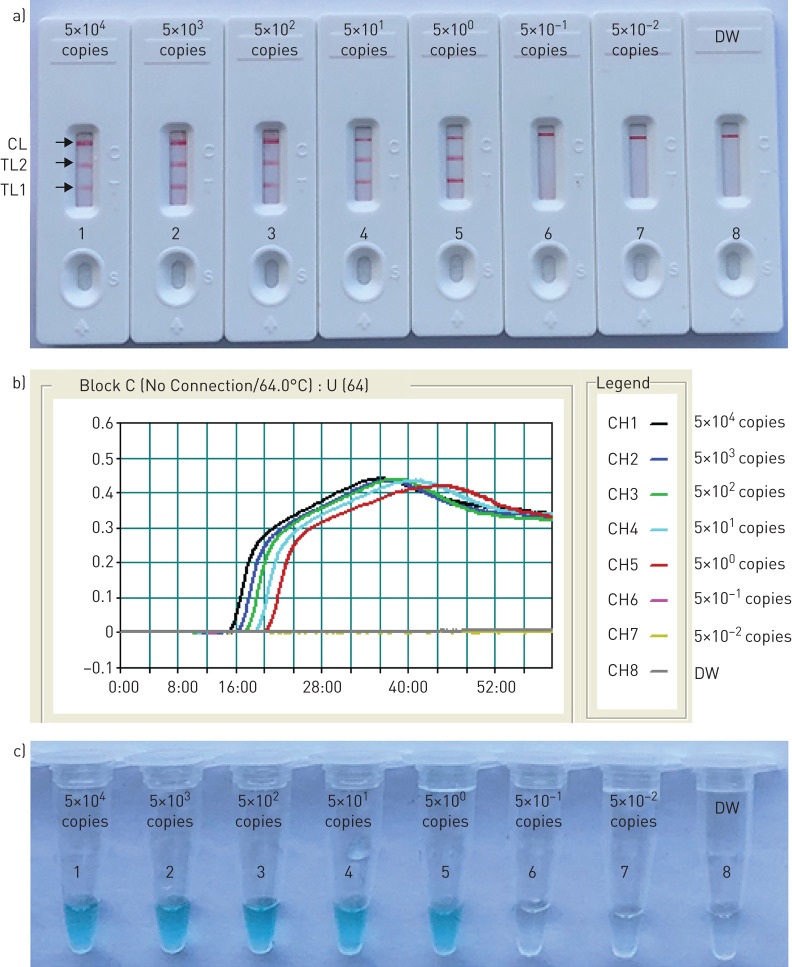
Sensitivity of coronavirus disease 2019 (COVID-19) reverse transcription multiple cross-displacement amplification nanoparticle-based biosensor assay (RT-MCDA-BS) using serially diluted plasmid templates. a) Biosensor applied for reporting the results; b) real-time turbidity applied for reporting the results; c) visual detection reagents applied for reporting the results. a) Biosensor, b) signals, c) tubes 1–8 represent the plasmid levels (of each of open reading frame 1a/b (F1ab)- and nucleoprotein (N)-plasmids) of 5×10^4^, 5×10^3^, 5×10^2^, 5×10^1^, 5×10^0^, 5×10^−1^, 5×10^−2^ copies per reaction and blank control (DW). The plasmid levels of 5×10^4^ to 5×10^0^ copies per reaction produced positive reactions.

As shown in figure 4, the COVID-19 RT-MCDA-BS assay can detect as few as five copies of either F1ab-plasmid or N-plasmid in a vessel. Of note, the F1ab and N genes of SARS-CoV-2 were simultaneously amplified correctly with their own primers in a one-step, single-tube reaction (figure 4a). The results obtained *via* biosensor were consistent with VDR reagent and real-time turbidity, while the VDR and real-time turbidity methods could not achieve multiple target detection (figure 4b and c). Most importantly, F1ab- and N-RT-MCDA assays also revealed a detection limit of five copies of target sequences in a reaction, which was consistent with the COVID-19 RT-MCDA assay (figure 4 and supplementary figures S4 and S5).

**FIGURE 5:**
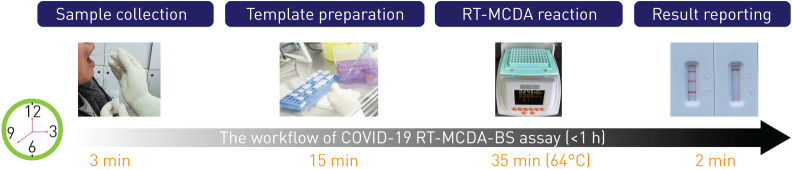
The workflow of coronavirus disease 2019 (COVID-19) reverse transcription multiple cross-displacement amplification nanoparticle-based biosensor assay (RT-MCDA-BS). Four steps, including sample collection (3 min), rapid template preparation (15 min), RT-MCDA reaction (35 min) and result reporting (within 2 min), are required for the COVID-19 RT-MCDA-BS assay, and the total procedure can be completed within 60 min.

The optimal reaction time required for the COVID-19 RT-MCDA-BS assay during the amplification stage was tested. The lowest template level (5×10^0^ copies of either F1ab- or N-plasmid displayed three red bands (TL1, TL2 and CL) when the isothermal reaction was conducted for 25 min at 64°C (supplementary figure S6). Therefore, for the clinical sample analysis, a reaction time of 35 min was recommended including the reverse transcription process (10 min). Thus, the whole diagnostic procedure of the COVID-19 RT-MCDA-BS technique, including sample collection (3 min), rapid template preparation (15 min), RT-MCDA reaction (35 min) and result reporting (<2 min), can be completed within 1 h (figure 5).

### Specificity of the COVID-19 RT-MCDA-BS assay

COVID-2019 RT-MCDA-BS assay specifically detected the positive control (containing 5×10^3^ copies of each of F1ab- and N-plasmids), while other target templates from non-COVID-19 viruses, bacteria and fungi were not detected (supplementary table S2). This suggests that the COVID-2019 RT-MCDA-BS assay has a very good specificity.

### Evaluation of COVID-19 RT-MCDA-BS assay in clinical samples

A total of 65 RNA samples from acute-phase and convalescent patients, which were initially detected using rRT-qPCR in the Guizhou province CDC in 2020, were tested to verify the feasibility of our assay. Our COVID-19 RT-MCDA-BS assay detected 22 (33.8%) positive results from different types of clinical specimens (*e.g.* faeces and nasal, anal and pharyngeal swabs), while rRT-PCR only found 20 (30.7%) positive results (supplementary table S3). The data suggest that our COVID-19 RT-MCDA-BS assay is more powerful in detecting COVID-19 patients, especially for those with very low virus loads. Furthermore, no positive results were obtained from these samples collected from non-COVID-19 patients (supplementary table S4).

## Discussion

With the unexpected occurrence of SARS-CoV-2 infection in Wuhan, the novel human coronavirus has since spread within China and to other regions/countries [15]. Hence, it is vital to develop adequate diagnosis techniques, which can provide a simple, rapid, reliable and easy-to-use strategy to detecting SARS-CoV-2 infection. Such diagnosis methods are required not only in countries where COVID-19 is spreading, but also in countries threatened by COVID-19. To tackle the pandemic efficiently, we successfully devised a novel method for diagnosis of COVID-19, termed COVID-19 RT-MCDA-BS assay.

The COVID-19 RT-MCDA-BS assay merges reverse transcription, cDNA isothermal amplification, and multiplex detection with a nano-biosensor, achieving the rapid diagnosis of COVID-19 in a one-step, single-tube reaction. Extremely simple instruments, such as a heating block, a water bath or even a thermos cup that can maintain a fixed temperature (64°C) for 30 min, is sufficient for COVID-19 RT-MCDA-BS test. Importantly, the biosensor provides an easy-to-use platform, which can objectively and visually indicate the COVID-19 RT-MCDA results, and eliminates the use of special colorimetric indicator such as electrophoresis and optical equipment. The whole diagnosis process of COVID-19 RT-MCDA-BS, including clinical sample collection (3 min), rapid template preparation (15 min), isothermal amplification (35 min) and result reporting (within 2 min), can be completed within 1 h. Herein, COVID-19 RT-MCDA-BS is an economical, rapid and technically simple method, which provides a measurement of practicality for “on-site”, field and clinical settings, especially for economically impoverished regions.

F1ab-MCDA and N-MCDA primer sets were designed, targeting 10 regions of F1ab and N genes, respectively, ensuring high selectivity for COVID-19 diagnosis. The specificity analysis demonstrated that COVID-19 RT-MCDA-BS could correctly diagnose the target pathogens with no false-positive results observed from non-SARS-CoV-2 templates, including bacterial, fungal and viral genomic templates (supplementary table S2). Furthermore, COVID-19 RT-MCDA-BS could simultaneously detect two target genes (F1ab and N) in a one-step isothermal reaction, which ensures reliability of COVID-19 diagnosis, eliminating the chance getting false-positive or false-negative results compared to other COVID-19 diagnosis methods that only detect a single molecular marker (*e.g.* F1ab biomarker).

A detection limit analysis demonstrated that COVID-19 RT-MCDA-BS is sensitive for the reliable detection of target sequences. The sensitivity of COVID-19 RT-MCDA-BS is very high with pure plasmid template down to five copies (each of F1ab-plasimd or N-plasmid) per reaction, completely in accordance with the detection results obtained from F1ab-RT-MCDA-BS and N-RT-MCDA-BS assays (figure 4 and supplementary figures S4 and S5). Compared to the singlex F1ab-RT-MCDA-BS and N-RT-MCDA-BS assays, COVID-19 RT-MCDA-BS did not show decreased or improved analytical sensitivity.

65 RNA samples isolated from faeces and nasal, anal and pharyngeal swabs of COVID-19 patients were examined to demonstrate the assay's feasibility in the clinic. The data suggest that the COVID-19 RT-MCDA-BS assay was able to analyse different types of clinical samples (supplementary table S3). 22 RNA samples tested positive according to the COVID-19 RT-MCDA-BS, whereas only 20 RNA samples were shown to be positive by rRT-PCR in the Guizhou province CDC. The findings suggest that our COVID-19 RT-MCDA-BS assay is more powerful in detecting COVID-19 patients, especially those with very low virus loads (supplementary table S3). The lower diagnosis rate of rRT-PCR might be explained by the presence of inhibitors which specifically affect the rRT-PCR assay, or the low copy numbers of the SARS-CoV-2 RNA templates, which is out of rRT-PCR assay's detection limit. Moreover, isothermal amplification-based assays, including MCDA-based methods (*e.g.* RT-MCDA-BS test), is less sensitive to various inhibitors, or is less affected by the presence of various salts from the sample buffer, or can tolerate the inhibitory effect of the large amounts of nucleic acids [16]. Particularly, no positive results were obtained from non-COVID-19 samples, which further validated the analytical specificity of the COVID-19 RT-MCDA-BS assay.

### Conclusion

We devised a method (COVID-19 RT-MCDA-BS) to diagnose COVID-19 using reverse transcription, multiplex detection and isothermal amplification coupled with a nanoparticle-based biosensor. Our data demonstrated that COVID-19 RT-MCDA-BS was a highly specific and sensitive diagnostic assay, and could be used as an attractive laboratory tool for diagnosis of COVID-19 in different types of clinical specimens. The diagnosis test of COVID-19 RT-MCDA-BS could be finished within 1 h, and did not rely on expensive reagents and apparatus. Collectively, its rapidity, low cost and ease of use make the COVID-19 RT-MCDA-BS method an ideal tool for use in field, primary care and clinical laboratories, especially for resource-poor settings.

## Supplementary material

10.1183/13993003.02060-2020.Supp1 **Please note:** supplementary material is not edited by the Editorial Office, and is uploaded as it has been supplied by the author.
Supplementary material ERJ-02060-2020.SUPPLEMENT

## Shareable PDF

10.1183/13993003.02060-2020.Shareable1 This one-page PDF can be shared freely online.
Shareable PDF ERJ-02060-2020.Shareable
