# Supplementary material for: Highly sensitive and specific diagnosis of COVID-19 by reverse transcription multiple cross-displacement amplification-labelled nanoparticles biosensor
Source: Eur Respir J. 2020 Dec 10;56(6):2002060. doi: 10.1183/13993003.02060-2020 (PMC7453731; doi:10.1183/13993003.02060-2020)
Supplement: Supplementary file 1 [file ERJ-02060-2020.SUPPLEMENT.pdf]

Supplementary Materials

Highly sensitive and specific diagnosis of coronavirus disease 19 (COVID-19) by reverse transcription multiple cross displacement amplification-labeled nanoparticles biosensor

Shijun Li [1], Weijia Jiang [1], Junfei Huang [1], Ying Liu [1], Lijuan Ren [1], Li Zhuang [1], Qinni Zheng[1], Ming Wang [1], Rui Yang [1], Yi Zeng [1], Yi Wang [2, 3*]

[1] Laboratory of Bacterial Infectious Disease of Experimental Center, Guizhou Provincial Center for Disease Control and Prevention, Guiyang 550004, P. R. China.

[2] Department of Respiratory Infection Disease, Beijing Pediatric Research Institute, Beijing Children's Hospital, Capital Medical University, National Center for Children's Health, Beijing 10045, P. R. China.

[3] Key Laboratory of Major Diseases in Children, Ministry of Education, Beijing Key Laboratory of Pediatric Respiratory Infection Disease, National Clinical Research Center for Respiratory Diseases, Beijing Children's Hospital, Capital Medical University, National Center for Children's Health, Beijing 10045, P. R. China.

*Correspondence: **Yi Wang**

Department of Respiratory Infection Disease, Beijing Pediatric Research Institute, Beijing Children's Hospital, National Center for Children's Health, No. 56, Nanlishi Road, Xicheng District, Beijing 100045, China.

E-mail: wildwolf0101@163.com

Tables

Table S1. The primers used in this study

Primers name [a]	Sequences and modifications [b]	Length [c]	Genes
F1ab-F1	5'-ACACTTAAAAACACAGTCTGTAC-3'	23 nt	
F1ab-F2	5'-AATTTAGCAAAACCAGCTACT-3'	21 nt	
F1ab -CP1	5'-GAAGCATGGGTTCGCGGAGTCGTCTGCGGTATGTGGAA-3'	38 mer	
F1ab -CP1*	5'-Dig-GAAGCATGGGTTCGCGGAGTCGTCTGCGGTATGTGGAA -3'	38 mer	
F1ab -CP2	5'-GTGCGGCACAGGCACTAGTATTATCATTGTAGATGTCAAAAGCC -3'	44 mer	
F1ab -C1	5'-GAAGCATGGGTTCGCGGAGT-3'	20 nt	F1ab
F1ab -C2	5'-GTGCGGCACAGGCACTAGTA-3'	20 nt	
F1ab -D1	5'-TGATCACAACTACAGCCAT-3'	19 nt	
F1ab -D2	5'-CTGATGTCGTATACAG-3'	16 nt	
F1ab -R1	5'-GATTGTGCATCAGCTGACT-3'	19 nt	
F1ab -R2	5'-GTTTGCGGTGTAAGTGCA-3'	18 nt	
N-F1	5'-GCTTCTACGCAGAAGGGA-3'	18 nt	
N-F2	5'-AGGCTTCTTAGAAGCCTCAG-3'	20 nt	
N-CP1	5'-CTACTGCTGCCTGGAGTTGAATTCTTCTCGTTCCTCATCACG-3'	42 mer	
N-CP1*	5'-FITC-CTACTGCTGCCTGGAGTTGAATTCTTCTCGTTCCTCATCACG-3'	42 mer	
N-CP2	5'-TGCTTGACAGATTGAACCAGCTGTGACAGTTTGGCCTTGTT-3'	41 mer	
N-C1	5'-CTACTGCTGCCTGGAGTTGAAT-3'	22 nt	N
N-C2	5'-TGCTTGACAGATTGAACCAGCT-3'	22 nt	
N-D1	5'-TCTTGAACTGTTGCGACTA-3'	19 nt	
N-D2	5'-TGAGAGCAAAATGTCTGGT-3'	19 nt	
N-R1	5'-CATTCTAGCAGGAGAAGTT-3'	19 nt	
N-R2	5'-ATGCTGCTCTTGCTTTGCT-3'	19 nt	

[a] F1ab, open reading frame 1a/b; N, nucleoprotein gene; F1ab-CP1*, 5'-labeled with Dig when used in RT-MCDA-BS assay; N-CP1*, 5'-labeled with FITC when used in RT-MCDA-BS assay.

[b] Dig, digoxigenin; FITC, fluorescein isothiocyanate.

[c] mer, monomeric unit; nt, nucleitide.

Table S2. The pathogens used in this study

**Strains**	**Serotypes (subtypes)**	**No. of strains**	**RT-LAMP-NBS**
Positive control	Unidentified	1	P
Coronavirus	HKU1	1	N
Influenza Virus A	H1N1	2	N
Influenza Virus A	H1N2	1	N
Influenza Virus A	H5	9	N
Influenza Virus A	H5N8	1	N
Influenza Virus A	H4N6	1	N
Influenza Virus A	H7N9	1	N
Influenza Virus B	Unidentified	3	N
Syncytial Virus	Unidentified	1	N
Human Adenovirus	Unidentified	2	N
Parainfluenza Virus	Unidentified	1	N
Bocavirus	Unidentified	1	N
Coxsackievirus	CAV16	1	N
Human enterovirus	EV71	1	N
Porcine circovirus	II	1	N
Swine fever virus	Unidentified	2	N
Lelysted Virus	Unidentified	2	N
Mycoplasma	Unidentified	1	N
Chlamydia	Unidentified	1	N
Lpneumophila	Unidentified	1	N
Pseudomonas aeruginosa	Unidentified	1	N
Staphylococcus aureus	Unidentified	1	N
Staphylococcus epidermidis	Unidentified	1	N
Staphylococcus saprophytics	Unidentified	1	N
Klebsiella pneumoniae	Unidentified	1	N
Neisseria meningitidis	Unidentified	1	N
Acinetobacter baumannii	Unidentified	1	N
Enterococcus faecalis	Unidentified	1	N
Enterococcus faecium	Unidentified	1	N
Shigella flexneria	Unidentified	1	N
Listeria monocytogenes	Unidentified	1	N
Staphylococcus suis	Unidentified	1	N
Bacillus cereus	Unidentified	1	N
Enterotoxigenic E. coli	Unidentified	1	N
Salmonella	Unidentified	1	N
Streptococcus pneumona	Unidentified	1	N
Vibrio parahemolyticus	Unidentified	1	N
Candida tropicalis	Unidentified	1	N

Cryptoccus neo formas	Unidentified	1	N
Candida albicans	Unidentified	1	N

Notes: Only positive control ($5x10^3$ copies each of F1ab-plasmid and np-plsmid) could be detected by the COVID-19 RT-MCDA-BS technique, indicating the extremely high selectivity of this method.

Table S3. Detection results of MCDA-BS for different types of samples

Sample number [a]	Sample type	Result of RT-PCR [b]	Ct value of RT-PCR [c]	Judgment result of MCDA-BS	Results of MCDA-BS		Patient status
					F1ab	N	
S1	Faeces	Positive	34.3	Positive	+	+	Convalescent
S2	Pharyngeal swab	Positive	36.6	Positive	+	+	Convalescent
S3	Nasal swab	Negative	—	Negative	—	—	Convalescent
S4	Faeces	Positive	35.4	Positive	+	_	Convalescent
S5	Faeces	Positive	33.7	Positive	+	+	Convalescent
S6	Faeces	Negative	—	Negative	—	—	Convalescent
S7	Faeces	Negative	—	Negative	—	—	Convalescent
S8	Faeces	Positive	36.2	Positive	+	—	Convalescent
S9	Pharyngeal swab	Negative	—	Negative	—	—	Convalescent
S10	Nasal swab	Negative	—	Negative	—	—	Convalescent
S11	Pharyngeal swab	Negative	—	Negative	—	—	Convalescent
S12	Anal swab	Negative	—	Negative	—	—	Convalescent
S13	Anal swab	Negative	—	Negative	—	—	Convalescent
S14	Faeces	Negative	—	Negative	—	—	Convalescent
S15	Faeces	Negative	—	Negative	—	—	Convalescent
S16	Faeces	Negative	—	Negative	—	—	Convalescent
S17	Pharyngeal swab	Negative	—	Negative	—	—	Convalescent
S18	Pharyngeal swab	Negative	—	Negative	—	—	Convalescent
S19	Faeces	Negative	—	Negative	—	—	Convalescent
S20	Anal swab	Negative	41.8	Positive	+	+	Convalescent
S21	Pharyngeal swab	Negative	—	Negative	—	—	Convalescent
S22	Anal swab	Negative	—	Negative	—	—	Convalescent
S23	Anal swab	Negative	—	Negative	—	—	Convalescent
S24	Anal swab	Negative	41.9	Positive	—	+	Convalescent
S25	Nasal swab	Negative	—	Negative	—	—	Convalescent
S26	Nasal swab	Negative	—	Negative	—	—	Convalescent
S27	Pharyngeal swab	Positive	35.2	Positive	+	+	Convalescent
S28	Pharyngeal swab	Positive	34.3	Positive	+	+	Convalescent
S29	Nasal swab	Negative	—	Negative	—	—	Convalescent
S30	Anal swab	Negative	—	Negative	—	—	Convalescent
S31	Nasal swab	Negative	—	Negative	—	—	Convalescent
S32	Anal swab	Negative	—	Negative	—	—	Convalescent
S33	Pharyngeal swab	Negative	—	Negative	—	—	Convalescent
S34	Anal swab	Negative	—	Negative	—	—	Convalescent
S35	Nasal swab	Negative	—	Negative	—	—	Convalescent
S36	Anal swab	Negative	—	Negative	—	—	Convalescent
S37	Nasal swab	Negative	—	Negative	—	—	Convalescent
S38	Anal swab	Negative	—	Negative	—	—	Convalescent

S39	Pharyngeal swab	Negative	—	Negative	—	—	Convalescent
S40	Anal swab	Negative	—	Negative	—	—	Convalescent
S41	Pharyngeal swab	Negative	—	Negative	—	—	Convalescent
S42	Anal swab	Negative	—	Negative	—	—	Convalescent
S43	Pharyngeal swab	Negative	—	Negative	—	—	Convalescent
S44	Anal swab	Negative	—	Negative	—	—	Convalescent
S45	Nasal swab	Negative	—	Negative	—	—	Convalescent
S46	Anal swab	Negative	—	Negative	—	—	Convalescent
S47	Pharyngeal swab	Negative	—	Negative	—	—	Convalescent
S48	Anal swab	Positive	33.3	Positive	+	+	Convalescent
S49	Pharyngeal swab	Negative	—	Negative	—	—	Convalescent
S50	Anal swab	Positive	34.5	Positive	+	+	Convalescent
S51	Pharyngeal swab	Negative	—	Negative	—	—	Convalescent
S52	Anal swab	Negative	—	Negative	—	—	Convalescent
S53	Pharyngeal swab	Negative	—	Negative	—	—	Convalescent
S54	Pharyngeal swab	Negative	—	Negative	—	—	Convalescent
S55	Pharyngeal swab	Positive	30.2	Positive	+	+	Acute phase
S56	Pharyngeal swab	Positive	32.4	Positive	+	—	Acute phase
S57	Pharyngeal swab	Positive	29.4	Positive	+	+	Acute phase
S58*	Pharyngeal swab	Positive	35.9	Positive	+	+	Acute phase
S59	Pharyngeal swab	Positive	25.4	Positive	+	+	Acute phase
S60	Pharyngeal swab	Positive	26.6	Positive	+	+	Acute phase
S61	Pharyngeal swab	Positive	29.3	Positive	+	+	Acute phase
S62	Pharyngeal swab	Positive	31.3	Positive	+	—	Acute phase
S63	Pharyngeal swab	Positive	28.4	Positive	+	+	Acute phase
S64	Pharyngeal swab	Positive	29.6	Positive	+	+	Acute phase
S65	Pharyngeal swab	Positive	30	Positive	+	+	Acute phase

[a] S58: this sample was collected from a asymptomatic individual.

[b] Positive: Ct <37; Negative: Ct>40 or no Ct.

[c] '—': no Ct.

Table S4. Detection results of COVID-19 RT-MCDA-BS method for non-COVID-19 samples

Sample number	Sample type	Result of COVID-19 RT-PCR	Result of COVID-19 RT-MCDA-BS	Confirmed pathogens*
S1	Pharyngeal swab	—	—	Influenza A virus
S2	Pharyngeal swab	—	—	Influenza A virus
S3	Pharyngeal swab	—	—	Influenza A virus
S4	Pharyngeal swab	—	—	Influenza A virus
S5	Pharyngeal swab	—	—	Influenza A virus
S6	Pharyngeal swab	—	—	Influenza A virus
S7	Pharyngeal swab	—	—	Influenza A virus
S8	Pharyngeal swab	—	—	Influenza A virus
S9	Pharyngeal swab	—	—	Influenza A virus
S10	Pharyngeal swab	—	—	Influenza A virus
S11	Pharyngeal swab	—	—	Influenza A virus
S12	Pharyngeal swab	—	—	Influenza A virus
S13	Pharyngeal swab	—	—	Influenza A virus
S14	Pharyngeal swab	—	—	Influenza A virus
S15	Pharyngeal swab	—	—	Influenza A virus
S16	Pharyngeal swab	—	—	Influenza A virus
S17	Pharyngeal swab	—	—	Influenza A virus
S18	Pharyngeal swab	—	—	Others
S19	Pharyngeal swab	—	—	Others
S20	Pharyngeal swab	—	—	Others
S21	Pharyngeal swab	—	—	Others
S22	Pharyngeal swab	—	—	Others
S23	Pharyngeal swab	—	—	Others
S24	Pharyngeal swab	—	—	Others
S25	Pharyngeal swab	—	—	Others
S26	Pharyngeal swab	—	—	Others
S27	Pharyngeal swab	—	—	Others
S28	Pharyngeal swab	—	—	Others
S29	Pharyngeal swab	—	—	Others
S30	Pharyngeal swab	—	—	Others
S31	Pharyngeal swab	—	—	Others
S32	Pharyngeal swab	—	—	Others
S33	Pharyngeal swab	—	—	Others
S34	Pharyngeal swab	—	—	Others
S35	Pharyngeal swab	—	—	Others
S36	Pharyngeal swab	—	—	Others
S37	Pharyngeal swab	—	—	Others
S38	Pharyngeal swab	—	—	Others
S39	Pharyngeal swab	—	—	Others
S40	Pharyngeal swab	—	—	Others
S41	Pharyngeal swab	—	—	Others

S42	Pharyngeal swab	—	—	Others
S43	Pharyngeal swab	—	—	Others
S44	Pharyngeal swab	—	—	Others
S45	Pharyngeal swab	—	—	Others
S46	Pharyngeal swab	—	—	Others
S47	Pharyngeal swab	—	—	Others
S48	Pharyngeal swab	—	—	Others
S49	Pharyngeal swab	—	—	Others
S50	Pharyngeal swab	—	—	Others
S51	Pharyngeal swab	—	—	Others
S52	Pharyngeal swab	—	—	Others
S53	Pharyngeal swab	—	—	Others
S54	Pharyngeal swab	—	—	Others
S55	Pharyngeal swab	—	—	Others
S56	Pharyngeal swab	—	—	Others
S57	Pharyngeal swab	—	—	Others
S58	Pharyngeal swab	—	—	Others
S59	Pharyngeal swab	—	—	Others
S60	Pharyngeal swab	—	—	Others
S61	Pharyngeal swab	—	—	Others
S62	Pharyngeal swab	—	—	Others
S63	Pharyngeal swab	—	—	Others
S64	Pharyngeal swab	—	—	Others
S65	Pharyngeal swab	—	—	Others
S66	Pharyngeal swab	—	—	Others
S67	Pharyngeal swab	—	—	Others
S68	Pharyngeal swab	—	—	Others
S69	Pharyngeal swab	—	—	Others
S70	Pharyngeal swab	—	—	Others
S71	Pharyngeal swab	—	—	Others
S72	Pharyngeal swab	—	—	Others
S73	Pharyngeal swab	—	—	Others
S74	Pharyngeal swab	—	—	Others
S75	Pharyngeal swab	—	—	Others
S76	Pharyngeal swab	—	—	Others
S77	Pharyngeal swab	—	—	Others
S78	Pharyngeal swab	—	—	Others
S79	Pharyngeal swab	—	—	Others
S80	Pharyngeal swab	—	—	Others
S81	Pharyngeal swab	—	—	Others
S82	Pharyngeal swab	—	—	Others
S83	Pharyngeal swab	—	—	Others
S84	Pharyngeal swab	—	—	Others
S85	Pharyngeal swab	—	—	Others

S86	Pharyngeal swab	—	—	Others
S87	Pharyngeal swab	—	—	Others
S88	Pharyngeal swab	—	—	Others
S89	Pharyngeal swab	—	—	Others
S90	Pharyngeal swab	—	—	Others
S91	Pharyngeal swab	—	—	Others
S92	Pharyngeal swab	—	—	Others
S93	Pharyngeal swab	—	—	Others
S94	Pharyngeal swab	—	—	Others
S95	Pharyngeal swab	—	—	Others
S96	Pharyngeal swab	—	—	Others
S97	Pharyngeal swab	—	—	Others
S98	Pharyngeal swab	—	—	Others
S99	Pharyngeal swab	—	—	Others
S100	Pharyngeal swab	—	—	Others
S101	Pharyngeal swab	—	—	Others
S102	Pharyngeal swab	—	—	Others
S103	Pharyngeal swab	—	—	Others
S104	Pharyngeal swab	—	—	Others
S105	Pharyngeal swab	—	—	Others
S106	Pharyngeal swab	—	—	Others
S107	Pharyngeal swab	—	—	Others
S108	Pharyngeal swab	—	—	Others
S109	Pharyngeal swab	—	—	Others
S110	Pharyngeal swab	—	—	Others
S111	Pharyngeal swab	—	—	Others
S112	Pharyngeal swab	—	—	Others
S113	Pharyngeal swab	—	—	Others
S114	Pharyngeal swab	—	—	Others
S115	Pharyngeal swab	—	—	Others
S116	Pharyngeal swab	—	—	Others
S117	Pharyngeal swab	—	—	Others
S118	Pharyngeal swab	—	—	Others

*Note： Influenza A virus includes H1N1, H3N2, H5N1,H7 subtype et, al; Others for Parainfluenza Virus, *Mycoplasma*, *Neisseria meningitidis* et, al.

Figures

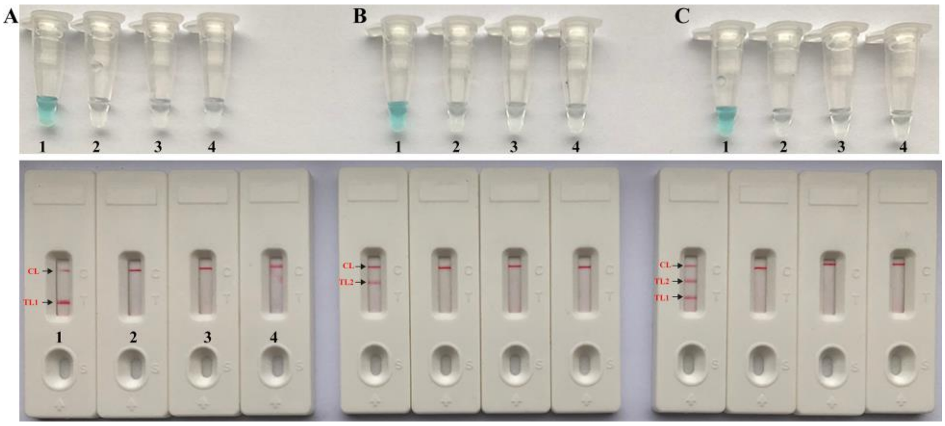

Figure S1. Confirmation and detection of F1ab- and N-RT-MCDA products

Color change of F1ab- (**A**, top row), N- (**B**, top row) and COVID-19 (**C**, top row) RT-MCDA tubes; BS applied for visual detection of F1ab- (**A**, bottom row), N- (**B**, bottom row) and COVID-19 (**C**, bottom row) RT-MCDA products. Tube A1 (BS A1), F1ab-RT-MCDA positive amplification (5×10^3 copies of F1ab-plasmids); Tube A2 (BS A2), F1ab-RT-MCDA negative amplification (H1N1); Tube A3 (BS A3), F1ab-RT-MCDA negative amplification (EV 71); Tube A4 (BS A4), Blank control (DW). Tube B1 (BS B1), N-RT-MCDA positive amplification (5×10^3 copies of N-plasmids); Tube B2 (BS B2), N-RT-MCDA negative amplification (H1N1); Tube B3 (BS B3), N-RT-MCDA negative amplification (EV 71); Tube B4 (BS B4), Blank control (DW). Tube C1 (BS C1), COVID-19 RT-MCDA positive amplification (5×10^3 copies of F1ab-plasmids and N-plasmids); Tube C2 (BS C2), COVID-19 RT-MCDA negative amplification (H1N1); Tube C3 (BS C3), COVID-19 RT-MCDA negative amplification (EV 71); Tube C4 (BS C4), Blank control (DW).

Note*: H1N1, Influenza H1N1 virus; EV 71, Human enterovirus 71; DW, Distilled water.

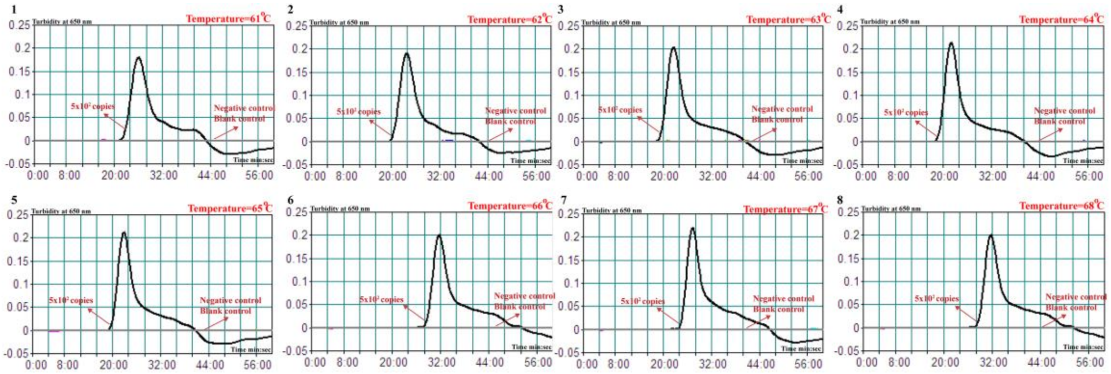

Figure S2. Optimal amplification temperature for F1ab-RT-MCDA primer set

The F1ab-RT-MCDA reactions for detection of F1ab gene of COVID-19 were monitored by real-time measurement of turbidity (LA320c). Turbidity of >0.1 was considered to be positive as the threshold value was 0.1. Eight kinetic graphs (1-8) were yielded at different temperatures (61-68°C, 1°C intervals) with target template at the level of 5×10^2 copies (F1ab-plamid) per reaction. The graphs from 63°C to 65°C showed faster amplification.

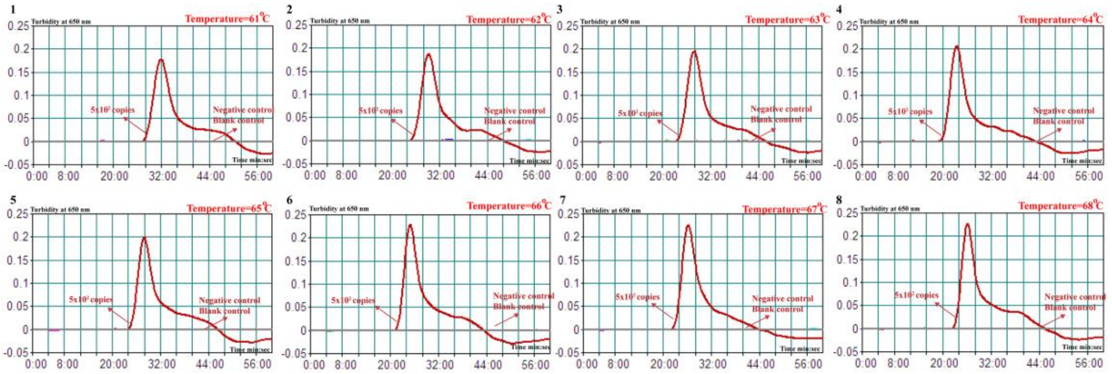

Figure S3. Optimal amplification temperature for N-RT-MCDA primer set

The N-RT-MCDA reactions for detection of N gene of COVID-19 were monitored by real-time measurement of turbidity (LA320c). Turbidity of >0.1 was considered to be positive as the threshold value was 0.1. Eight kinetic graphs (1-8) were yielded at different temperatures (61-68°C, 1°C intervals) with target template at the level of 5×10^{2} copies (N-plasmid) per reaction. The graphs from 63°C to 65°C showed faster amplification.

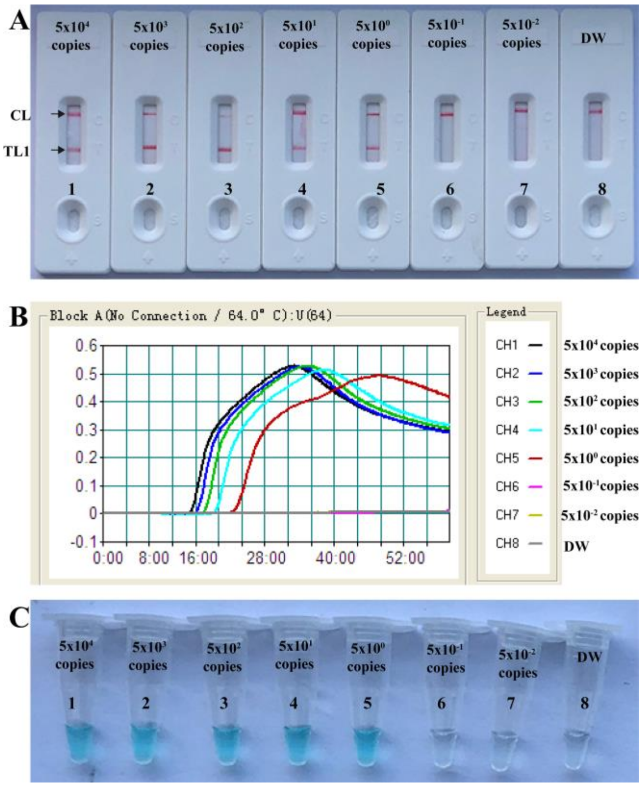

Figure S4. Analytical sensitivity of F1ab-RT-MCDA-BS assay using serially diluted F1ab-plasmid templates

A, BS applied for reporting the results; **B**, Real-time turbidity applied for reporting the results; **C**, VDR applied for reporting the results. BS (A)/Signals (B)/Tubes (C) 1-8 represented the F1ab-plasmid levels of 5×10^{4}, 5×10^{3}, 5×10^{2}, 5×10^{1}, 5×10^{0}, 5×10^{-1}, 5×10^{-2} copies per reaction and blank control (DW). The plasmid levels of 5×10^{4} to 5×10^{0} copies per reaction produced the positive reactions.

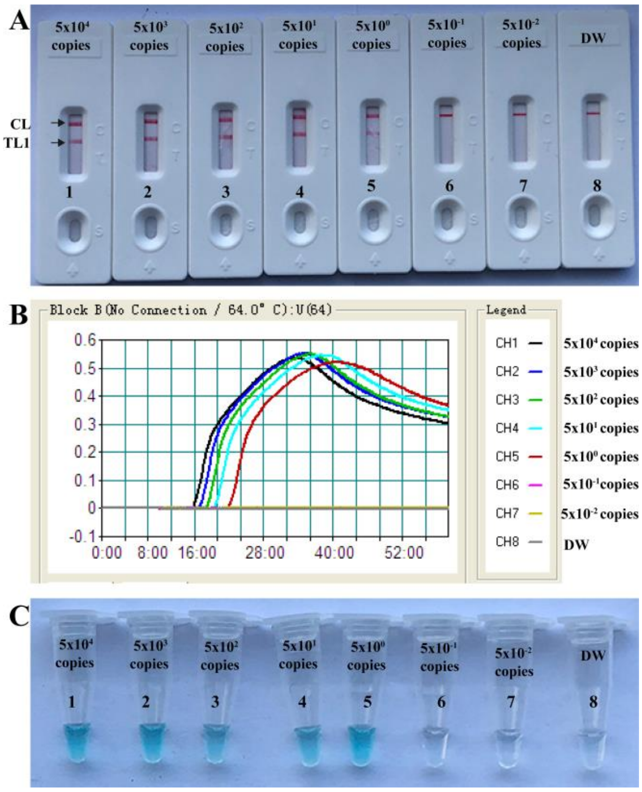

Figure S5. Analytical sensitivity of N-RT-MCDA-BS assay using serially diluted N-plasmid templates

A, BS applied for reporting the results; **B**, Real-time turbidity applied for reporting the results; **C**, VDR applied for reporting the results. BS (A)/Signals (B)/Tubes (C) 1-8 represented the N-plasmid levels of 5×10^4, 5×10^3, 5×10^2, 5×10^1, 5×10^0, 5×10^{-1}, 5×10^{-2} copies per reaction and blank control (DW). The plasmid levels of 5×10^4 to 5×10^0 copies per reaction produced the positive reactions.

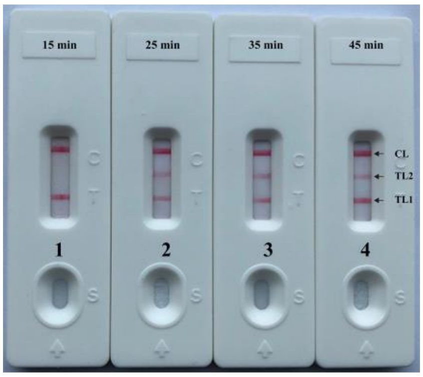

Figure S6. The optimal duration of time required for COVID-19 RT-MCDA-BS assay

Four reaction times (**A**, 15 min; **B**, 25 min; **C**, 35 min; and **D**, 45 min) were tested and compared at 64 ^{o}C. COVID-19 RT-MCDA reactions were conducted using the LoD level of templates (5×10^0 copies of each of F1ab-plasmid and N-plasmid), and the templates at the LoD level could be detected when the RT-MCDA reaction only lasted for 25 min (**Biosensor 2**).
