# Supplementary material for: Highly sensitive and specific diagnosis of COVID-19 by reverse transcription multiple cross-displacement amplification-labelled nanoparticles biosensor
Source: Eur Respir J. 2020 Dec 10;56(6):2002060. doi: 10.1183/13993003.02060-2020 (PMC7453731; doi:10.1183/13993003.02060-2020)
Supplement: Supplementary file 2 [file ERJ-02060-2020.Shareable.pdf]

SHAREABLE PDF

Highly sensitive and specific diagnosis of COVID-19 by reverse transcription multiple cross-displacement amplification-labelled nanoparticles biosensor

Shijun Li[1], Weijia Jiang[1], Junfei Huang[1], Ying Liu[1], Lijuan Ren[1], Li Zhuang[1], Qinni Zheng[1], Ming Wang[1], Rui Yang[1], Yi Zeng[1] and Yi Wang[2,3]

Affiliations: [1]Laboratory of Bacterial Infectious Disease of Experimental Center, Guizhou Provincial Center for Disease Control and Prevention, Guiyang, P.R. China. [2]Dept of Respiratory Disease, Beijing Pediatric Research Institute, Beijing Children's Hospital, Capital Medical University, National Center for Children's Health, Beijing, P.R. China. [3]Key Laboratory of Major Diseases in Children, Ministry of Education, Beijing Key Laboratory of Pediatric Respiratory Infection Disease, National Clinical Research Center for Respiratory Diseases, Beijing Children's Hospital, Capital Medical University, National Center for Children's Health, Beijing, P.R. China.

Correspondence: Yi Wang, Dept of Respiratory Disease, Beijing Pediatric Research Institute, Beijing Children's Hospital, Capital Medical University, National Center for Children's Health, No. 56, Nanlishi Road, Xicheng District, Beijing 100045, China. E-mail: wildwolf0101@163.com

@ERSpublications
We devised a novel method (COVID-19 RT-MCDA-BS) to diagnose COVID-19. Its rapidity, low cost and ease of use make the method an ideal tool for use in field, primary and clinical laboratories, especially for resource-poor settings. https://bit.ly/3j08CU2

Cite this article as: Li S, Jiang W, Huang J, *et al*. Highly sensitive and specific diagnosis of COVID-19 by reverse transcription multiple cross-displacement amplification-labelled nanoparticles biosensor. *Eur Respir J* 2020; 56: 2002060 [https://doi.org/10.1183/13993003.02060-2020].

This single-page version can be shared freely online.

ABSTRACT

Background: The ongoing outbreak of the novel human coronavirus severe acute respiratory syndrome coronavirus 2 (SARS-CoV-2) (also known as 2019-nCoV) has become a global health concern. Rapid and easy-to-use diagnostic techniques are urgently needed to diagnose SARS-CoV-2 infection.

Methods: We devised a reverse transcription multiple cross-displacement amplification (RT-MCDA) coupled with a nanoparticle-based biosensor assay (RT-MCDA-BS) for rapid, sensitive and specific diagnosis of coronavirus disease 2019 (COVID-19). Two primer sets were designed to target the open reading frame 1a/b and nucleoprotein gene of SARS-CoV-2. A total of 183 clinical samples, including 65 patients with COVID-19 infection and 118 patients with other pathogen infections were used to testify the assay's feasibility. Diagnosis results were reported visually using the biosensor.

Findings: The assay designed was performed using a simple instrument which could maintain the reaction in a constant temperature at 64°C for only 35 min. The total COVID-19 RT-MCDA-BS test procedure could be finished within 1 h. The COVID-19 RT-MCDA-BS could detect down to five copies of target sequences. Among 65 clinical samples from the COVID-19 patients, 22 (33.8%) positive results were obtained from faeces, nasal, pharyngeal and anal swabs *via* COVID-19 RT-MCDA-BS assay, while real-time reverse transcription-PCR assay only detected 20 (30.7%) positive results in these samples. No positive results were obtained from clinical samples with non-COVID-19 infections.

Interpretation: COVID-19 RT-MCDA-BS was a rapid, reliable, low-cost and easy-to-use assay, which

Copyright ©ERS 2020. This version is distributed under the terms of the Creative Commons Attribution Non-Commercial Licence 4.0.

could provide an attractive laboratory tool to diagnose COVID-19 in multiple clinical specimens, especially for field, clinic laboratories and primary care facilities in resource-poor settings.
